# Simulations of pattern dynamics for reaction-diffusion systems via SIMULINK

**DOI:** 10.1186/1752-0509-8-45

**Published:** 2014-04-11

**Authors:** Kaier Wang, Moira L Steyn-Ross, D Alistair Steyn-Ross, Marcus T Wilson, Jamie W Sleigh, Yoichi Shiraishi

**Affiliations:** 1 School of Engineering, The University of Waikato, Private Bag 3105, Hamilton 3240, New Zealand; 2 Waikato Clinical School, The University of Auckland, Waikato Hospital, Hamilton 3204, New Zealand; 3 Department of Product Science and Technology, Gunma University, 29-1 Hon-cho, Ohta-shi, Gunma 373-0052, Japan

**Keywords:** Simulink modelling, Brusselator model, Cortical model, Turing–Hopf pattern

## Abstract

**Background:**

Investigation of the nonlinear pattern dynamics of a reaction-diffusion system almost always requires numerical solution of the system’s set of defining differential equations. Traditionally, this would be done by selecting an appropriate differential equation solver from a library of such solvers, then writing computer codes (in a programming language such as C or Matlab) to access the selected solver and display the integrated results as a function of space and time. This “code-based” approach is flexible and powerful, but requires a certain level of programming sophistication. A modern alternative is to use a graphical programming interface such as Simulink to construct a data-flow diagram by assembling and linking appropriate code blocks drawn from a library. The result is a visual representation of the inter-relationships between the state variables whose output can be made completely equivalent to the code-based solution.

**Results:**

As a tutorial introduction, we first demonstrate application of the Simulink data-flow technique to the classical van der Pol nonlinear oscillator, and compare Matlab and Simulink coding approaches to solving the van der Pol ordinary differential equations. We then show how to introduce space (in one and two dimensions) by solving numerically the partial differential equations for two different reaction-diffusion systems: the well-known Brusselator chemical reactor, and a continuum model for a two-dimensional sheet of human cortex whose neurons are linked by both chemical and electrical (diffusive) synapses. We compare the relative performances of the Matlab and Simulink implementations.

**Conclusions:**

The pattern simulations by Simulink are in good agreement with theoretical predictions. Compared with traditional coding approaches, the Simulink block-diagram paradigm reduces the time and programming burden required to implement a solution for reaction-diffusion systems of equations. Construction of the block-diagram does not require high-level programming skills, and the graphical interface lends itself to easy modification and use by non-experts.

## Background

Natural systems exhibit an amazing diversity of patterned structures in both living and nonliving systems [1], such as animal coats (e.g., zebra stripes, leopard spots and fish spirals), chemicals in a gel [2], laser light in a cavity [3], charges on the surface of a semiconductor [4], ecological balance between two species [5] and neuronal activations in human cortex [6]. Generally, there exist two kinds of natural patterns [7]: 

• Thermodynamic equilibrium-based, such as crystal structures in inorganic chemistry and spontaneously emergent organic polymer patterns. The forming mechanisms of these patterns have been extensively studied and well-explained via thermodynamics and statistical physics: When a system’s temperature decreases, its entropy and the Gibbs free energy accordingly become smaller (systems tend to move towards a thermodynamic equilibrium where the Gibbs free energy reaches a minimum). Thus the principle of minimum energy allows the system to form certain spatial structures.

• Thermodynamics far-from-equilibrium, such as examples mentioned at the beginning of this section. These patterns emerge away from thermodynamic equilibrium, thus thermodynamic theory is not applicable. The understanding of these patterns may require application of kinetic theories.

Pattern dynamics research focuses on universal pattern-forming mechanisms for the latter case. Away from thermodynamic equilibrium, some experimentally observed 2D spatial patterns have been reported: Rayleigh-Bénard convection patterns [8]; reaction-diffusion Turing patterns [9]; Faraday-wave patterns [10]; vibratory granular patterns [11]; slime-mold spiral patterns [12] and Ca^2+^ spiral waves in Xenopus laevis eggs [13]. Although these patterns are different in the spatiotemporal scales or detailed pattern-forming mechanisms, they have similar structures.

More than half a century ago, British mathematician Alan Turing strove to understand the spontaneous processes generating these structures. In his famous 1952 paper [14], he proposed a reaction-diffusion model explaining potential mechanism for animal coats: At a certain stage of embryonic development, the reaction and diffusion between molecules, known as *morphogens*, and other reactors, lead to the breaking of symmetry of the homogeneous state. The morphogens spontaneously evolve to a non-uniform state, leading to the unique textures seen on animal skin. The key pattern-forming idea in Turing’s paper is the system’s spontaneous symmetry-breaking mechanism, also known as a *Turing bifurcation* (spatial instability).

However, for nearly 30 years Turing’s paper drew little attention for two reasons: morphogens had not been found in biological systems; negative chemical concentrations are permitted in Turing’s model, but are not accepted by chemists. From the late 1960s, a Brussels team led by Ilya Prigogine (winner of 1977 Nobel prize in chemistry) was endeavouring to prove Turing’s theory. Prigogine developed a reaction-diffusion model, *Brusselator*[15], to show the existence of Turing patterns that obey the rules of chemical kinetics. The Brusselator is a mathematical representation of the interaction between two *morphogens*, a *reactor* and an *inhibitor*, competitively reacting in time and spreading in space, which could give rise to a symmetry-breaking transition bifurcating from a homogeneous to patterned state, either stationary in a spatial (Turing) structure or in a temporal (Hopf) oscillation [15]. The Brusselator model further revealed the “secret” of Turing patterns: a coupling between nonlinear reaction kinetics and distinct diffusion rates such that the inhibitor should diffuse more rapidly than the activator [16].

This Brusselator proposed activator–inhibitor interaction is now a well known universal principle explaining regular pattern formation in chemical [17-19], ecological [5,20] and physical [21,22] systems, as well as biological systems [23,24].

As pattern-forming systems essentially consist of coupled differential equations, the simulated patterns are time- and space-dependent solutions of the differential equations. The MATLAB built-in fourth-order Runge-Kutta solver ode45 and custom Euler methods are common ways to integrate differential systems. The implementation of these algorithms are well explained and demonstrated in many studies [25-28]. For example, in Yang’s book [25], at the end of Part II Yang presents a piece of concise MATLAB code for efficiently simulating simple reaction-diffusion systems. With some modifications, Yang’s programs can be used to simulate pattern formation in a wide range of applications of nonlinear reaction-diffusion equations. Some of these examples are discussed in detail in Part III of Yang’s book. Alternative to MATLAB, there are other options for pattern simulations such as MEREDYS[29], implemented in the Java programming language, interpreting the NEUROML model description language [30]. Besides, there are other programming environments applicable to modelling pattern formations such as COMSOL[31] and MODELICA[32].

Although it is efficient to solve differential equations in MATLAB or other programming platforms, their code script-based pattern simulations require high-level programming skills to tune the model parameters in order to examine the pattern dynamics; this limits their uptake by non-programmers. A few attempts for graphic-based pattern simulations have been made in the last decades. For example, READY[33] is an ideal program for getting started with reaction-diffusion systems. It runs fast (taking advantage of multi-core architectures), is easy to use (graphic-based) and runs on multiple platforms. In addition, there are other Java applets allowing easy pattern simulations such as Gray-Scott Simulator [34], showing the Gray-Scott pattern [35]: phenomena in grids of points that are connected “amorphously”. This closely models the development of a biological system of living cells. Similarly, Lidbeck has created another Java application [36] with extensive presets and options, and even allows 3-D of pattern simulations. However, these graphical user interface (GUI)-based softwares designed mainly for demonstrations of specific (pre-loaded) models. READY supports custom models but is written in READY-specific codes. So technically, READY is still a coding-based platform with a GUI interface. In the community of pattern dynamicists, there may be a demand for a software platform circumventing the programming burden required to implement numerical simulations of biologically-relevant pattern-forming systems.

The aim of this paper is to introduce SIMULINK modelling for simulating pattern dynamics. SIMULINK, an add-on product to MATLAB, provides an interactive, graphical environment for simulating and analysing dynamic systems. It enables modelling via a graphical programming language based on block diagrams. SIMULINK has been used extensively in engineering field [37] such as control theory and digital signal processing for multidomain simulation [38] as well as model-based design [39]. Besides, SIMULINK users have extended its applications in other areas such as medical device prototyping [40], heat transfer modelling [41,42] and chemical reactions [43].

The present paper introduces the application of SIMULINK to pattern simulation studies, and it is organised as follows. We start, for pedagogical reasons, with a brief demonstration of the SIMULINK in modelling differential equations. Then, we construct the well-known Brusselator model in SIMULINK. By extending the Brusselator modelling strategies, we construct a SIMULINK-based human cortical model [44] (developed by the authors’ team) that incorporates the pattern-forming essentials while retaining neurophysiological accuracy: the cortical model comprises interacting populations of excitatory and inhibitory neurons which communicate via chemical (neurotransmitter-controlled) and electrical (gap-junction) synapses. In the model, the interaction between chemical kinetics and electrical diffusion allow for the emergence of a comprehensive range of patterns, which may be of direct relevance to clinically observed brain dynamics such as epileptic seizure EEG spikes [6], deep-sleep slow-wave oscillations [45] and cognitive gamma-waves [46,47].

Finally, we examine the Brusselator and cortical model pattern dynamics. These simulation results are compared with the theoretical predictions.

## Methods

Consider a generalised reaction-diffusion system for two reacting and diffusive species *U* and *V* of the form:

(1) $$\begin{array}{l} \frac{\partial U}{\partial t}=f_{U}(U,V)+D_{U}\nabla^{2}U\\ \frac{\partial V}{\partial t}=f_{V}(U,V)+D_{V}\nabla^{2}V \end{array}$$

The diffusion constant *D*_*U*,*V*_ [with units (length)^2^/time] is an important parameter indicative of the diffusion mobility. For a multi-component system, the higher the diffusivity, the faster the species diffuse into each other. Here, *f*_*U*,*V*_(*U*,*V*) is typically a nonlinear function of concentrations *U* and *V*.

Solving a pattern-forming system in the form of Eq. (1) requires interpreting the differential operators for time *∂*/*∂t* and space ∇^2^. In the following example, we first model the van der Pol oscillator in SIMULINK to explain how we interpret the differential operator on time.

### Van der Pol oscillator

The van der Pol oscillator was originally developed by the Dutch electrical engineer and physicist Balthasar van der Pol [48]. The van der Pol oscillator was the first mathematical model proposed for the heartbeat, and it has also been used to simulate brain waves [49,50]:

(2) $$\frac{d^{2}x}{dt^{2}}-\mu \left(1-x^{2}\right)\frac{\text{dx}}{\text{dt}}+x=0$$

We wish to solve this equation for the case *μ* = 1 with initial conditions *x*(0) = 2 and *dx*/*dt* = 0 at *t* = 0. The traditional way to solve a second-order differential equation is to convert to a pair of coupled first-order differential equations:

(3) $$\begin{array}{l} \dot{x}=y\\ \dot{y}=\mu \left(1-x^{2}\right)y-x \end{array}$$

We would now integrate these equations with time using the MATLAB numerical integrator ode45. This helps to form the link with the integration in SIMULINK.

**Figure 1:**
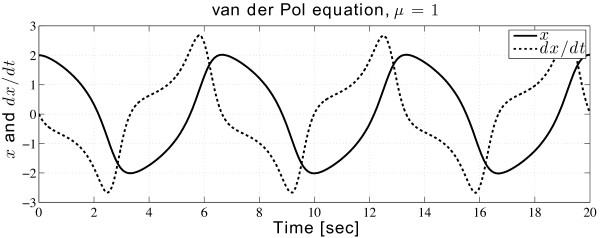
**Solution of the van der Pol equation, produced via MATLAB code sheet.** Program running time: 0.384 s in variable time-step. Simulation platform (same for all simulations in this paper): MATLAB R2013a, MAC OS X 10.9.1, XCODE 5.0.2; CPU 2.4 GHz Intel Core i7, memory 8 GB 1600 MHz DDR3.

We code the first-order van der Pol equations into a MATLAB function^a^ as follows:

To solve Eq. (3), we specify the coefficient *μ*, the initial conditions and the time-span over which the integration is to proceed; then pass these values, along with the name of the van der Pol function, to the Runge-Kutta solver ode45:

The calculated results are plotted in Figure 1.

**Figure 2:**
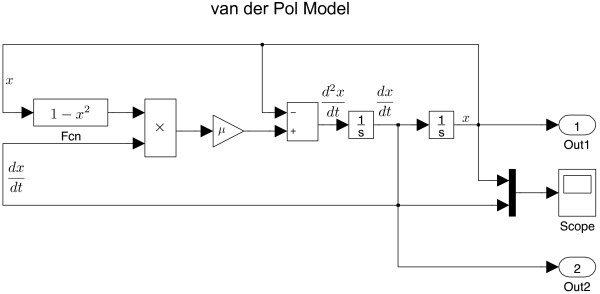
**SIMULINK ** **built-in example for the van der Pol model called by the MATLAB ** **command ****vdp****.**

Alternatively, we may use the SIMULINK construction of Eq. (2), as shown in Figure 2.

At a first glance, the interface for SIMULINK is completely different from the code sheet. In SIMULINK, all calculating elements are displayed by blocks. We select blocks from the SIMULINK library, then connect them to build a model.

The basic principle to model a differential equation in SIMULINK is to find the input and output of an integrator. Since we have:

(4) $$\int \left[\int \frac{d^{2}x}{dt^{2}}\text{dt}\right]\text{dt}=\int \frac{\text{dx}}{\text{dt}}\text{dt}=x$$

then it follows that for a second-order differential equation, we need at least two integrators. As seen in Figure 2, we first place an integrator block (the left block labelled with $\frac{1}{s}$) to process the inner integration of Eq. (4): $\int \frac{d^{2}x}{dt^{2}}\text{dt}$. The output of this integrator reads *dx*/*dt*, which is sent into the second integrator (the right block labelled with $\frac{1}{s}$). We assume the integrated *x* is known, thus being used to construct the input of the left integrator block, which is equivalent to $\frac{d^{2}x}{dt^{2}}$ with the form:

(5) $$\frac{d^{2}x}{dt^{2}}=\mu \left(1-x^{2}\right)\frac{\text{dx}}{\text{dt}}-x$$

The product block (labelled with ×) in Figure 2 combines (1 - *x*^2^) and *dx*/*dt*. The result is amplified by a gain (triangle block, valued *μ*), then passed through a function block where *x* is subtracted. Here, the RHS of Eq. (5) is constructed.

Modelling a differential equation in SIMULINK requires forming a closed loop, where the integrated variables are fed back into the system. Evolution proceeds until reaching the desired final time. The scope block shows the real-time output of the two integrators; the scope can be placed anywhere to monitor the response of a sub-system. The Out1 and Out2 terminals send outputs of two integrators to the MATLAB workspace for further analysis. The results of this SIMULINK model are exactly the same as shown in Figure 1.

**Figure 3:**
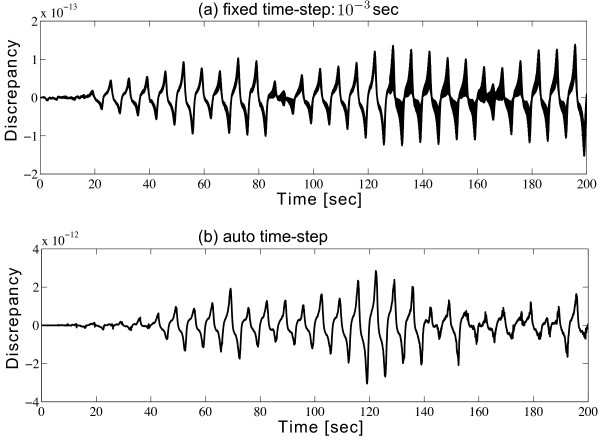
**Comparision for the van der Pol equation solved from SIMULINK and MATLAB code sheet.** Discrepancy over time for the solution of Eq. (2) is calculated from two modelling methods: SIMULINK and MATLAB code sheet. Both methods use **(a)** fixed time-step 10^-3^ s; or **(b)** auto time-step. Program running time for SIMULINK in fixed and auto time-steps are respective 1.3410 and 0.7404 s; for MATLAB the corresponding time are 17.3952 and 9.4569 s.

Both MATLAB and SIMULINK allow fixed or self-adaptive (i.e., auto) time-steps for the Runge-Kutta solver^b^. Figure 3 shows that the discrepancy between MATLAB and SIMULINK Runge-Kutta solvers in either fixed or auto time-step mode are sufficiently small (< 10^-10^). Consequently, we can see that the accuracy of the model simulation does not depend on the modelling platform since MATLAB and SIMULINK share the same integration algorithm to solve differential equations. However, modelling in SIMULINK is more straightforward and intuitive, and requires less programming skill than the MATLAB code sheet. The original mathematical equations can be converted into SIMULINK by matching its pattern with SIMULINK blocks directly. Moreover, in SIMULINK, by simply adding more blocks, or replacing blocks, a new model is able to be built in a very short time. SIMULINK may be an ideal tool to efficiently perform the simulations of a mathematical model. In the next section, we will extend the SIMULINK modelling method to describe a Brusselator system considering both its temporal and spatial evolutions on 1-D and 2-D Cartesian grids.

Readers should be aware of the choice of an appropriate differential solver for a specific problem, depending on the stiffness of differential equations. Applying a wrong solver may lead to either unstable solution or exceptional computation time. However, it is practically difficult to identify the stiffness of a differential model, thus one should try at least two different solvers, and compare the results. If they concur, i.e. give the same solution, they are likely to be correct. As suggested by MATLAB help file, it is worthwhile to try ode45 first since it is the most widely used method. For pattern-forming systems, we can also compare the numerical solution with the theoretical prediction to identify the applicability of the solver. For the demonstrated Brusselator and cortical models, ode45 and ode23 both work well and give rise to similar result; moreover, the numerical solutions match well with the theoretical predictions in emergent patterns (see **Results and discussion** section). So we choose ode45 solver to integrate the differential-equation models in this paper.

### Brusselator model

The Brusselator model describes the competition of two chemical species in a chemical reaction, and is the simplest reaction-diffusion system capable of generating complex spatial patterns. The competition between two reactors and the introduction of diffusion satisfy the key requirements for pattern formation [14]. The pattern dynamics of the Brusselator has been comprehensively examined in de Wit [15] and other researchers’ work [51-54]. Here, our purpose is to introduce the SIMULINK modelling strategies.

#### SIMULINK version of Brusselator model

The simplest form of the model reads [55],

(6) $$\begin{array}{l} \frac{\partial }{\partial t}X=A-(B+1)X+X^{2}Y+D_{X}\nabla^{2}X\\ \frac{\partial }{\partial t}Y=\text{BX}-X^{2}Y+D_{Y}\nabla^{2}Y \end{array}$$

where *X* and *Y* denote concentrations of activator and inhibitor respectively; *D*_*X*_ and *D*_*Y*_ are diffusion constants; *A* is a constant and *B* is a parameter that can be varied to result in a range of different patterns.

The LHS of Eq. (6) is a partial derivative on time since *X* and *Y* are functions of both time and space. At the RHS, the spatial derivative is represented by a Laplacian operator ∇^2^. In the numerical simulation, the spatial dimension of the model is discretised into a grid by using the finite difference method. In the two-dimensional system the Laplacian with respect to the concentration field *U* in the node (*i*,*j*) is calculated along the *x* and *y* directions simultaneously:

(7) $$\nabla^{2}U_{i,j}\approx \frac{\Delta_{x}^{2}U_{i,j}}{h_{x}^{2}}+\frac{\Delta_{y}^{2}U_{i,j}}{h_{y}^{2}}$$

where

(8) $$\begin{array}{l} \Delta_{x}^{2}U_{i,j}=U_{i+1,j}-2U_{i,j}+U_{i-1,j};\\ \Delta_{y}^{2}U_{i,j}=U_{i,j+1}-2U_{i,j}+U_{i,j-1} \end{array}$$

The *h*_*x*,*y*_ demonstrators in Eq. (7) are the respective *x* and *y* grid spacings; they define the spatial resolution. Assuming *h* ≡ *h*_*x*_ = *h*_*y*_ (i.e., a square grid), the discrete Laplacian operation in a one-dimensional Cartesian coordinates along the *y*-axis has the form:

(9) $$\nabla_{\text{1D}}^{2}U_{i,j}\approx \frac{U_{i,j+1}-2U_{i,j}+U_{i,j-1}}{h^{2}};$$

for the two-dimensional case, we have

(10) $$\begin{array}{l} \nabla_{\text{2D}}^{2}U_{i,j}\approx \frac{U_{i+1,j}+U_{i-1,j}-4U_{i,j}+U_{i,j+1}+U_{i,j-1}}{h^{2}} \end{array}$$

In SIMULINK, we initialise the Brusselator model as a column vector consisting of a 60 × 1 grid (spatial resolution = 1 cm/grid-point) for the one-dimensional case; or as a 60 × 60 grid for the two-dimensional case. Grid edges are joined to give toroidal boundaries.

The Laplacian operator $\nabla_{\text{1D}}^{2}$ in Eq. (9) is implemented as a circular convolution of the 3 × 1 second-difference kernel $L_{\text{1D}}^{y}$ acting along the *y*-axis:

(11) $$\begin{array}{l} \nabla_{\text{1D}}^{2}\approx L_{\text{1D}}^{y}=\frac{1}{h_{y}^{2}}\left[\begin{array}{c} 1\\ -2\\ 1 \end{array}\right] \end{array}$$

The two-dimensional Laplacian operator $\nabla_{\text{2D}}^{2}$ in Eq. (10) is built up from the sum of two orthogonal *L*_1D_ operators:

(12) $$\begin{array}{l} \nabla_{\text{2D}}^{2}\approx L_{\text{2D}}=\frac{1}{h_{x}^{2}}\left[\begin{array}{ccc} 0 & 1 & 0\\ 0 & -2 & 0\\ 0 & 1 & 0 \end{array}\right]+\frac{1}{h_{y}^{2}}\left[\begin{array}{ccc} 0 & 0 & 0\\ 1 & -2 & 1\\ 0 & 0 & 0 \end{array}\right]=\frac{1}{h^{2}}\left[\begin{array}{ccc} 0 & 1 & 0\\ 1 & -4 & 1\\ 0 & 1 & 0 \end{array}\right] \end{array}$$

where we have again assumed a square grid so that *h*_*x*_ = *h*_*y*_ = *h*.

**Figure 4:**
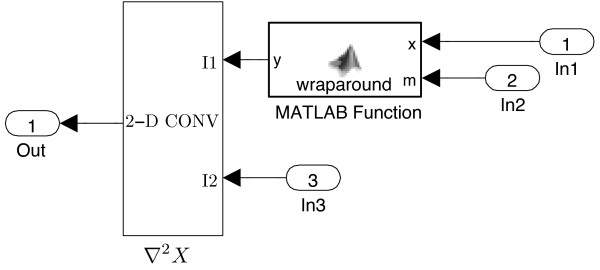
**SIMULINK modelling of the convolution with toroidal boundaries.** The spatial derivative ∇^2^*X* approximates to the discrete convolution of *X* given by the kernal *L*. *X* will be fed into the port In1, the kernel *L* enters In2 and In3.

In SIMULINK, the 1-D or 2-D Laplacian operator with toroidal boundaries is processed through two blocks: The “wrap-around” and “2-D CONV” (can process both 1-D and 2-D convolutions). The “wrap-around” block wraps the input matrix on both axes to allow a valid convolution in the “2-D CONV” block against the Laplacian kernel *L* to return the cyclic convolution. We created a subsystem to compute the convolution, as shown in Figure 4.

In Figure 4, the custom block labelled “wraparound” contains codes extracted from the convolve2() function^c^.

The reason we introduce the custom block is that the SIMULINK built-in 2-D CONV block provides only the “valid” (non-flux) boundary condition, and cannot handle periodic boundaries.

**Figure 5:**
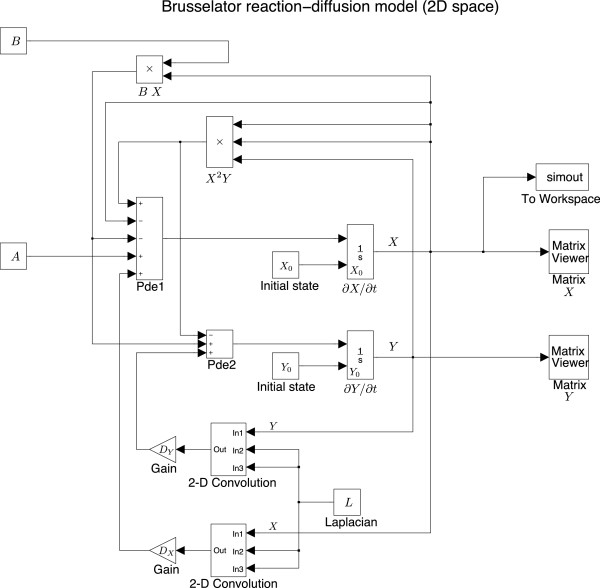
**SIMULINK ** **construction of the Brusselator model.**

Following the ideas of modelling the van der Pol oscillator, we can easily convert Eq. (6) to SIMULINK blocks, seen in Figure 5.

### SIMULINK versions of Waikato cortical model equations

The authors’ team at the University of Waikato (New Zealand) has developed a mean-field cortical model which encapsulates the essential neurophysiology of the cortex, while remaining computationally cost efficient [44]. The model envisions the cortex as a continuum in which pools of neurons are linked via chemical and electrical (gap-junction) synapses.

#### Model background

The Waikato cortical model has a rich history of development: The model is based on early work by Liley *et al.*[56], with enhancements motivated by Rennie *et al.*[57] and Robinson *et al.*[58]; and has been recently extended to include electrical synapses (also referred as gap junctions) [44,47] supplementing neural communications via chemical synapses.

**Figure 6:**
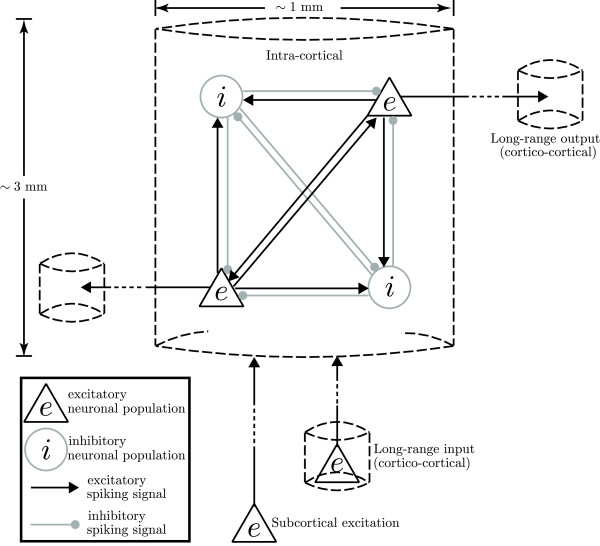
**Schematic representation of the connective topology within a cortical macrocolumn.** Only four of the ∼100,000 neurons are shown. The shapes of neurons are determined based on their appearances under microscope: triangles are excitatory (pyramidal) neurons; circles are inhibitory neurons.

The Waikato cortical modelling assumptions are: 

1. Cortical element is the “macrocolumn” containing ∼100,000 neurons.

2. There are only two distinct kinds of cortical neurons: 85% excitatory, and 15% inhibitory neurons. “Excitatory” and “inhibitory” are classified according to their effects on other neurons.

3. There are three isotropic neuronal interactions: cortico-cortical (long-range from other macrocolumns), intra-cortical (short-range within macrocolumn) and connections from subcortical structures such as the thalamus and brain-stem. A macrocolumn with diameter ∼1 mm and depth ∼3 mm is sketched in Figure 6. We further assume that inhibitory connections via their short axons are locally restricted within a macrocolumn. In contrast, the axons from excitatory neurons are often longer and extensively branched, having length ranging from centimetres to several meters (e.g., in a giraffe’s neck), allowing exclusively excitatory cortico-cortical connections. Both excitatory and inhibitory connections are permitted in local neuron connections.

4. Neurons exchange information via both chemical and electrical (gap-junction) synapses. Gap-junctions are more abundant within inhibitory populations, while being rare within excitatory neuronal populations.

The authors’ team first introduced gap-junctions into the cortical model in the paper *Gap-junction mediate large-scale Turing structures in a mean-field cortex driven by subcortical noise*[44]. The cortical model is expected to exhibit similar dynamics to a chemical reaction-diffusion system: The interaction between the excitatory and inhibitory neurons is analogous to to the competition between the activator and inhibitor of the chemical reaction-diffusion model, e.g. the Brusselator. The gap-junction strength between inhibitory neurons also plays the same role as the diffusion terms in the Brusselator, which allows a spatial evolution of the patterns. Consequently, it is reasonable that the cortical model exhibits similar pattern dynamics for those seen in the chemical reaction-diffusion system.

#### Model equations

The neuron populations deliver spike flux *ϕ*_*ab*_ from sources *Q*_*a*_ at a distance following damped wave equations [58]:

(13) $$\left[{\left(\partial /\partial t+v\Lambda_{\text{ab}}\right)}^{2}-v^{2}\nabla^{2}\right]\phi_{\text{ab}}=v^{2}\Lambda_{\text{ab}}^{2}Q_{a}$$

The subscript *ab* is read “*a* → *b*”, the connection from the presynaptic neuron type *a* to postsynaptic neuron type *b*. *a*,*b* can be either *e* (excitatory) or *i* (inhibitory). *Q*_*a*_ is the spike-rate flux, which is a mapping from membrane voltage *V*_*a*_ to population-averaged firing rates:

(14) $$Q_{a}=\frac{Q_{a}^{\text{max}}}{1+e^{-C(V_{a}-\theta_{a})/\sigma_{a}}}$$

The total input flux arriving at the post-synapse is given as,

(15) $$M_{\text{ab}}=\underset{\text{long-range}}{\underset{︸}{N_{\text{eb}}^{\alpha }\;\phi_{\text{eb}}^{\alpha }\;}}+\underset{\text{local}}{\underset{︸}{N_{\text{ab}}^{\beta }\;\phi_{\text{ab}}^{\beta }\;}}+\underset{\text{subcortical}}{\underset{︸}{\phi_{\text{eb}}^{\text{sc}}\;}}$$

where superscripts *α* and *β* distinguish long-range and local chemical synaptic inputs; $N_{\text{eb}}^{\alpha }$ and $N_{\text{ab}}^{\beta }$ are the number of such input connections. The cortex is driven by subcortical noise which enters the intra-cortex. Subcortical excitation $\phi_{\text{eb}}^{\text{sc}}$ is modelled as small-amplitude spatiotemporal Gaussian-distributed white noise superimposing on a background excitatory “tone” $\langle \phi_{\text{eb}}^{\text{sc}}\rangle$ whose constant level is set via a subcortical drive scale-factor *s*:

(16) $$\phi_{\text{eb}}^{\text{sc}}(t)=s\langle \phi_{\text{eb}}^{\text{sc}}\rangle +\xi_{\text{eb}}(t)\sqrt{s\langle \phi_{\text{eb}}^{\text{sc}}\rangle }$$

where *ξ*_*eb*_ is the Gaussian-distributed white-noise sources being delta-correlated in time and space. Inclusion of white-noise stimulation is motivated by physiological evidence that the cortex requires a continuous background “wash” of input noise to function normally.

The total input flux Φ_*ab*_ is the temporal convolution of the dendrite impulse response *H*(*t*) with the input flux *M*_*ab*_:

(17) $$\begin{array}{ll} \;\Phi_{\text{ab}} & =(\text{dendrite response})⊗(\text{input flux})\\ & =H_{b}(t)⊗M_{\text{ab}}(t)\\ & =\int_{0}^{t}H_{b}(t-t^{\prime })\;M_{\text{ab}}(t^{\prime })dt^{\prime } \end{array}$$

where the dendrite dynamics are modelled by the alpha-function impulse response

(18) $$H_{b}(t)=\gamma_{b}^{2}te^{-\gamma_{b}t}$$

**Figure 7:**
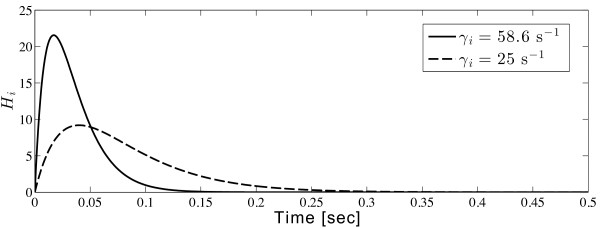
**Cortical IPSP (inhibitory post-synaptic potential) response (inhibitory alpha-function) curve.** With delays in the inhibitory postsynaptic response (reduction of the inhibitory rate-constant *γ*_*i*_), the occurrence of the IPSP peak will be delayed. Here, the position of the peak shifts from 0.017 to 0.04 s by reducing *γ*_*i*_ from 58.6 to 25 s^-1^.

Reducing *γ*_*i*_ is equivalent to increasing the time-to-peak (1/ *γ*_*i*_) for the hyperpolarising GABA (gamma-aminobutyric acid) response, as indicated in Figure 7.

By taking derivatives, Eq. (17) becomes

(19) $${\left(\frac{\partial }{\partial t}+\gamma_{a}\right)}^{2}\Phi_{\text{ab}}=\gamma_{a}^{2}M_{\text{ab}}(t)$$

Therefore, the flux received by target neuronal populations are:

(20a) $${\left(\frac{\partial }{\partial t}+\gamma_{e}\right)}^{2}\Phi_{\text{eb}}=\left[N_{\text{eb}}^{\alpha }\phi_{\text{eb}}^{\alpha }+N_{\text{eb}}^{\beta }Q_{e}+\phi_{\text{eb}}^{\text{sc}}\right]\gamma_{e}^{2}$$

(20b) $${\left(\frac{\partial }{\partial t}+\gamma_{i}\right)}^{2}\Phi_{\text{ib}}=N_{\text{ib}}^{\beta }Q_{i}\gamma_{i}^{2}$$

Finally, those incoming spikes perturb the soma resting potentials:

(21) $$\begin{array}{l} \tau_{b}\;\frac{\partial V_{b}}{\partial t}\;=\;V_{b}^{\text{rest}}-V_{b}+\underset{chemical synapses}{\underset{︸}{\rho_{e}\psi_{\text{eb}}\Phi_{\text{eb}}+\rho_{i}\psi_{\text{ib}}\Phi_{\text{ib}}\;}}+\underset{electrical synapses}{\underset{︸}{D_{1,2}\nabla^{2}V_{b}}} \end{array}$$

We write *D*_*bb*_ as the diffusive-coupling strength between electrically adjoined *e* ↔ *e*, *i* ↔ *i* neuron pairs. To simplify the notation, we write (*D*_1_,*D*_2_) ≡ (*D*_*ee*_,*D*_*ii*_).

Symbol definitions for the cortical model are summarised in Table 1.

**Table 1:** Symbol definitions for the cortical model

**Symbols**	**Description**	**Value**	**Unit**
Λ_*e*,*b*_	Inverse-length scale for *e*→*b* axonal connection	4	cm^-1^
*v*	Axonal conduction speed	140	cm s^-1^
$Q_{e,i}^{\text{max}}$	Maximum firing rate	30, 60	s^-1^
*θ*_*e*,*i*_	Sigmoid threshold voltage	-58.5, -58.5	mV
*σ*_*e*,*i*_	Standard deviation for threshold	3, 5	mV
*C*	Constant	$\pi /\sqrt{3}$	
$N_{\text{eb}}^{\alpha }$	Long-range *e* → *b* axonal connectivity	2000	-
$N_{\text{eb},\text{ib}}^{\beta }$	Local *e* → *b*, *i* → *b* axonal connectivity	800, 600	-
*γ*_*e*_	Excitatory rate-constant	170	s^-1^
*γ*_*i*_	Inhibitory rate-constant	50	s^-1^
$\langle \phi_{\text{eb}}^{\text{sc}}\rangle$	*e*→*b* tonic flux entering from subcortex	300	s^-1^
*τ*_*e*,*i*_	Neuron time constant	0.04, 0.04	s
$V_{e,i}^{\text{rev}}$	Reversal potential at dendrite	0, -70	mV
$V_{e,i}^{\text{rest}}$	Neuron resting potential	-64, -64	mV
$\Delta V_{e,i}^{\text{rest}}$	Offset to resting potential	1.5, 0	mV
*ρ*_*e*_	Excitatory synaptic gain	1.00×10^-3^	mV s
*ρ*_*i*_	Inhibitory synaptic gain	-1.05×10^-3^	mV s
*D*_2_	*i* ↔ *i* gap-junction diffusive coupling strength	0–2.0	cm^2^
*D*_1_	*e* ↔ *e* gap-junction diffusive coupling strength	*D*_2_/100	cm^2^

#### SIMULINK versions of Waikato cortical model equations

Let us first list the mathematical equations for the Waikato cortical model and examine their characteristics. 

• The cortico-cortical equation

$$\left[{\left(\frac{\partial }{\partial t}+v\Lambda_{\text{eb}}\right)}^{2}-v^{2}\nabla^{2}\right]\phi_{\text{eb}}^{\alpha }={\left(v\Lambda_{\text{eb}}\right)}^{2}Q_{e}$$

  can be arranged by collecting temporal derivatives to the LHS:

(22) $$\frac{\partial^{2}}{\partial t^{2}}\phi_{\text{eb}}^{\alpha }+2v\Lambda_{\text{eb}}\frac{\partial }{\partial t}\phi_{\text{eb}}^{\alpha }\;=\;v^{2}\nabla^{2}\phi_{\text{eb}}^{\alpha }-v^{2}\Lambda^{2}\phi_{\text{eb}}^{\alpha }+{\left(v\Lambda_{\text{eb}}\right)}^{2}Q_{e}$$

• The intra-cortical equations

$$\begin{array}{l} {\left(\frac{\partial }{\partial t}+\gamma_{e}\right)}^{2}\Phi_{\text{eb}}=\left[N_{\text{eb}}^{\alpha }\phi_{\text{eb}}^{\alpha }+N_{\text{eb}}^{\beta }Q_{e}+\phi_{\text{eb}}^{\text{sc}}\right]\gamma_{e}^{2}\\ \;{\left(\frac{\partial }{\partial t}+\gamma_{i}\right)}^{2}\Phi_{\text{ib}}=\left[N_{\text{ib}}^{\beta }Q_{i}\right]\gamma_{i}^{2} \end{array}$$

  have different RHS, but their LHS are in the same mathematical pattern:

(23) $${\left(\frac{\partial }{\partial t}+\gamma \right)}^{2}\Phi =\frac{\partial^{2}}{\partial t^{2}}\Phi +2\gamma \frac{\partial }{\partial t}\Phi +\gamma^{2}\Phi$$

  We can move the term *γ*^2^Φ to the RHS of the intra-cortical equations, then the LHS of the intra-cortical equations have the expression:

(24) $$\frac{\partial^{2}}{\partial t^{2}}\Phi +2\gamma \frac{\partial }{\partial t}\Phi$$

  which is similar to the LHS of the cortico-cortical equation.

• The soma equation

$$\tau_{b}\frac{\partial V_{b}}{\partial t}=V_{b}^{\text{rest}}-V_{b}+(\rho_{e}\psi_{\text{eb}}\Phi_{\text{eb}}+\rho_{i}\psi_{\text{ib}}\Phi_{\text{ib}})+D_{\text{bb}}\nabla^{2}V_{b}$$

  can be re-arranged as

(25) $$\begin{array}{l} \frac{\partial V_{b}}{\partial t}\;=\;\frac{1}{\tau_{b}}\left[\;V_{b}^{\text{rest}}\;-\;V_{b}\;+\;(\rho_{e}\psi_{\text{eb}}\Phi_{\text{eb}}\;+\;\rho_{i}\psi_{\text{ib}}\Phi_{\text{ib}})\;+\;D_{\text{bb}}\nabla^{2}V_{b}\;\right] \end{array}$$

Following the ideas of SIMULINK modelling in van der Pol oscillator, we need two integrator blocks for Eqs. (22) and (24), and two convolution processing for Eqs. (22) and (25).

**Figure 8:**
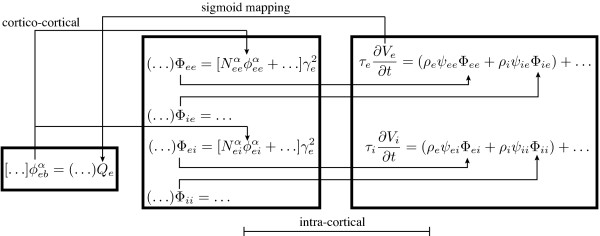
Flux flows for the Waikato cortical model between its cortico-cortical, intra-cortical and soma equation subsystems.

The strategy for modelling a large system is to focus on its subsystems first, then connect them together. The Waikato cortical model has three major parts: cortico-cortical, intra-cortical and soma equations. Figure 8 shows how neuronal fluxes are transferred from one to another: cortico-cortical flux $\phi_{\text{eb}}^{\alpha }$ is delivered to the long-range targets Φ_*ee*_ and Φ_*ei*_; intra-cortical flux Φ_*ee*_ and Φ_*ei*_, Φ_*ie*_ and Φ_*ii*_ merge into the soma equations. The output of the soma *V*_*e*_ is connected to source neurons to form the closed loop through the excitatory sigmoid function:

$$Q_{e}=\frac{Q_{e}^{\text{max}}}{1+e^{-C(V_{e}-\theta_{e})/\sigma_{e}}}$$

In following sections, we detail the SIMULINK implementation of the three subsystems (drawn as three blocks in Figure 8) of the cortical model.

##### 

**Figure 9:**
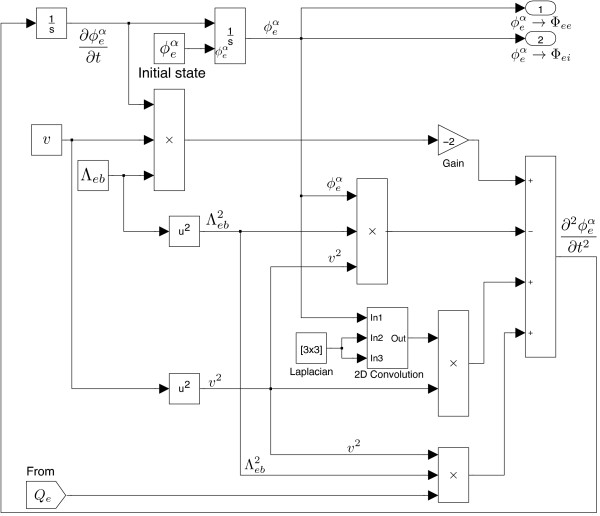
**SIMULINK-based cortico-cortical wave-equation.**

**Cortico-cortical flux** The SIMULINK based cortico-cortical block (see Figure 9) is converted from Eq. (22). The flux-source *Q*_*e*_ is a mapping from the excitatory soma voltage sent via the Goto block, to the firing-rate received via the From block. After two integrations, signals will be passed to the excitatory synapses via port-1 (upper right corner) and inhibitory synapses via port-2 in the intra-cortex.

##### 

**Figure 10:**
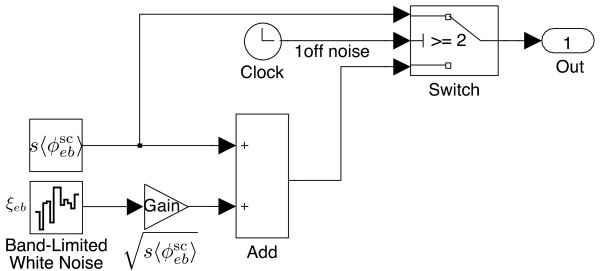
**SIMULINK-based sub-cortical flux.**

**Intra-cortical flux** In SIMULINK modelling, we divide Eq. (16) into two parts: the constant (i.e. dc-level) excitatory background $s\langle \phi_{\text{eb}}^{\text{sc}}\rangle$ and the one-off kick $\sqrt{s\langle \phi_{\text{eb}}^{\text{sc}}\rangle }\xi_{\text{eb}}$. As illustrated in Figure 10, we use a Clock block to count the iteration step. Once the counter is above one, the “switch” will turn off the kick, allowing only the constant excitation to enter the intra-cortex (removing the Clock block would allow on-going noise stimulus from the subcortex). The 2-D spatial white noise are generated by the Band-Limited White Noise block.

**Figure 11:**
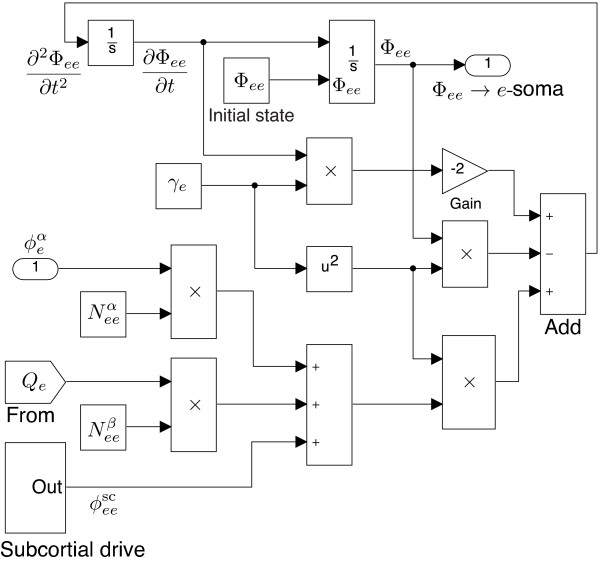
**SIMULINK-based*e*** **→** ***e* post-synaptic flux** **Φ**_***ee***_** for the intra-cortex.**

The intra-cortical model describes how post-synaptic fluxes evolve over time. In Figure 11, the local fluxes (input via the From block) along with the long-range fluxes (from input-1 at the left, labelled as $\phi_{e}^{\alpha }$) and subcortical drive are summed, then filtered at the post-synaptic dendrite, thus forming the post-synaptic fluxes Φ_*ee*_ at the output port-1 (upper right corner). The Φ_*ee*_ and Φ_*ei*_ flux models have symmetric structure.

**Figure 12:**
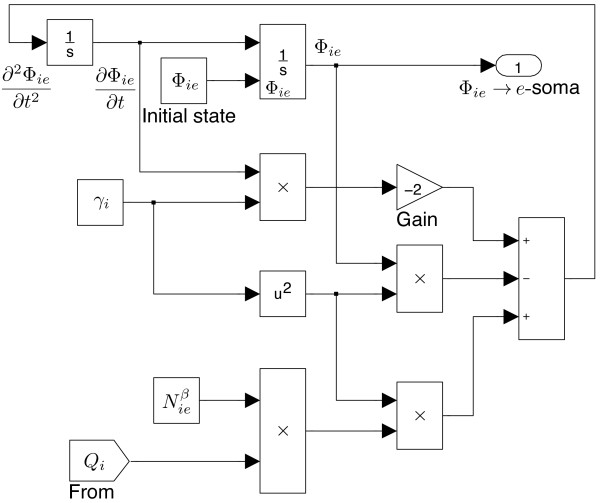
**SIMULINK-based** ***i*** **→** ***e* post-synaptic flux** **Φ**_***ie***_** for the intra-cortex.**

We assume that the cortico-cortical fibres are exclusively excitatory, thus there are no long-range inhibitory fluxes entering into the soma. Figure 12 shows that the local inhibitory fluxes Φ_*ie*_ come from local source *Q*_*i*_ only. The Φ_*ie*_ and Φ_*ii*_ models also have symmetric structure.

##### Soma voltage

**Figure 13:**
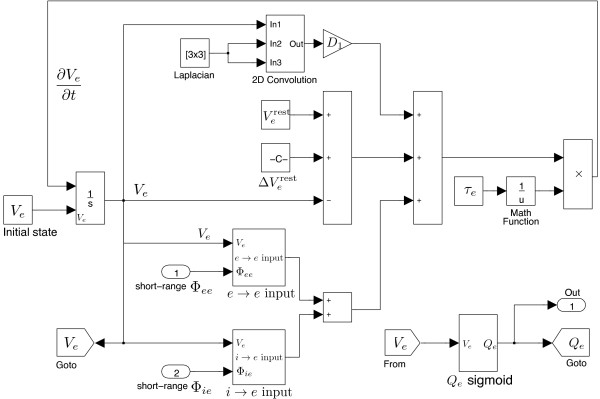
**SIMULINK-based excitatory soma equation.**

Figure 13 presents the soma model of the excitatory neuronal group. The short-range fluxes are accumulated at the soma, from ports 1 and 2. The soma voltage *V*_*e*_ is converted to firing-rate *Q*_*e*_ locally in this sub-model (block labelled with *Q*_*e*_ sigmoid), then fed back into the cortico-cortical and intra-cortical models.

**Figure 14:**
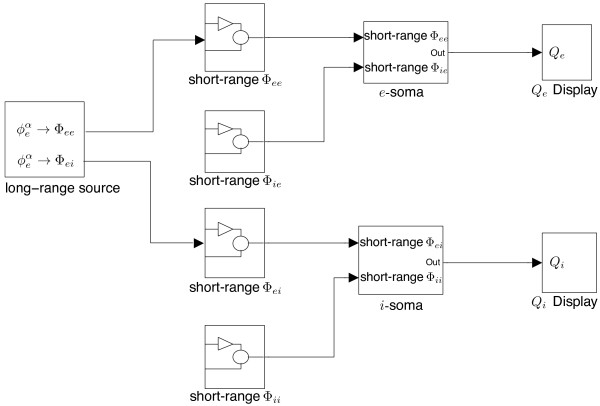
**Overview of the SIMULINK ** **implementation of the Waikato cortical equations.**

**Figure 15:**
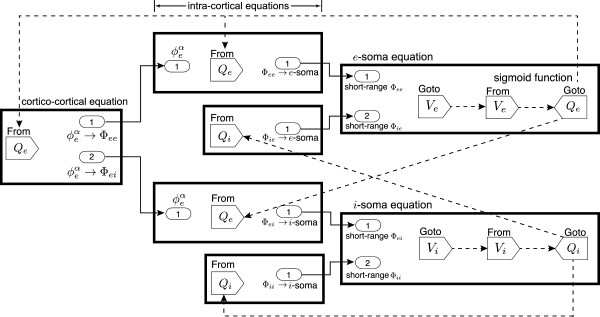
**Detailed connection diagram for the SIMULINK-based Waikato cortical equations.** Solid arrow: direct connection; dashed arrow: Goto–From connection.

Finally, we connect all subsystems to form the completed Waikato cortical model, as illustrated in Figure 14. It follows the flux flow-chart of Figure 8, with the detailed SIMULINK block connections shown in Figure 15. We argue that such model-based-design is an advantage for representing differential equations in SIMULINK. Although SIMULINK is useful for rapid prototyping, the SIMULINK implementation of the cortical model runs slower than our pre-coded Euler integration^d^ since it is time-consuming to interpret the MATLAB function wrapround (see Figure 4) in SIMULINK. A 60×60 grid (side length 20 cm) 5-s cortical simulation takes ∼10 s via MATLAB Euler integration (fixed time-step 0.8 ×10^-3^ s), while ∼40 s via SIMULINK (auto time-step and in accelerator mode). Thus, it is recommended to avoid using MATLAB functions in SIMULINK unless necessary.

## Results and discussion

### Brusselator pattern simulations in 1-D space

**Figure 16:**
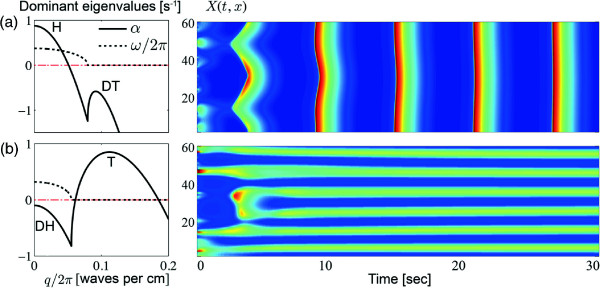
**Brusselator simulation in 1-D space.** Left column: dispersion curve of real (*α*) and imaginary (*ω*) parts of dominnat eigenvalues predicting the emergent pattens for two sets of parameters: **(a)** *A* = 2.5, *B* = 9, *D*_*X*_ = 7, *D*_*Y*_ = 10; **(b)** *A* = 2, *B* = 4.8, *D*_*X*_ = 2, *D*_*Y*_ = 10. H: Hopf mode with *α* > 0,*ω* > 0 at wavenumber *q* = 0; T: Turing mode with *α* > 0,*ω* = 0 at *q* ≠ 0; DT: damped Turing with *α* < 0 at *q* ≠ 0; DH: damped Hopf with *α* < 0 at *q* = 0. Right column: one-dimensional Brusselator model of length 60 cm with periodic boundary condition evolves in time running rightwards during 30 s. Colour indicates the local concentration of the reactant: [red] high concentration, [blue] low concentration.

Before simulation, we first predict the pattern dynamics of the Brusselator model. Following the work by Steyn-Ross *et al.*[44], we applied a linear stability analysis (LSA) [59] by examining the sign of the real and imaginary parts of the dominant eigenvalue (wavenumber *q* dependent) *σ*_*q*_ = *α*(*q*) + *iω*(*q*) derived from the Jacobian matrix at a steady-state. LSA states that the stability of the steady-state: *α*(*q*) > 0 leads to unstable steady-state; *α*(*q*) < 0 leads to stable steady-state. The scaled imaginary part *ω*(*q*)/2*π* predicts the oscillation frequency of the pattern at the wavenumber *q*. Considering different combinations of the sign of *α* and *ω*, there are four main classes of instability, summarised in Table 2. Following these rules, the LSA shown in Figure 16(a) and (b) predict respectively a Hopf and Turing instability. The simulation in the upper panel shows a homogeneous oscillations, in the bottom panel exhibits a frozen spatial structure. Both simulations are in good agreement with the LSA predictions.

**Table 2:** The onsets of four main classes of instability

			**Pattern stability**	
**Bifurcation**	**Critical wavenumber**	**Eigenvalue (*σ***_***q***_ ***= α + iω*)**	**Spatially**	**Temporally**
Turing	*q* ≠ 0	*σ*_*q*_ = 0	Unstable	Stable
Hopf	*q* = 0	*α* = 0, *ω* ≠ 0	Stable	Unstable
Turing–Hopf	*q* ≥ 0	*α* = 0, *ω* ≠ 0 at *q* = 0	Unstable	Unstable
Wave	*q* ≠ 0	*α* ≥ 0, *ω* > 0	Wave instability	

### Brusselator pattern simulations in 2-D space

Brusselator simulation in a 2-D space has more varieties than in a 1-D space. These 2-D patterns will have unique spatial Turing structures that we cannot infer from the LSA. To precisely predict the Turing pattern dynamics, we derived the amplitude equations [54] for the Brusselator model at the onset of a Turing instability. The amplitude equation describes a reduced form of a reaction-diffusion system yet still retains its essential dynamical features. By approximating the analytic solution, the amplitude equation allows precise predictions of the pattern dynamics when the system is near a bifurcation point. In simple words, the amplitude equations may offer a guidance for model parameter settings corresponding to specific patterns, e.g., Turing pattern textures.

**Figure 17:**
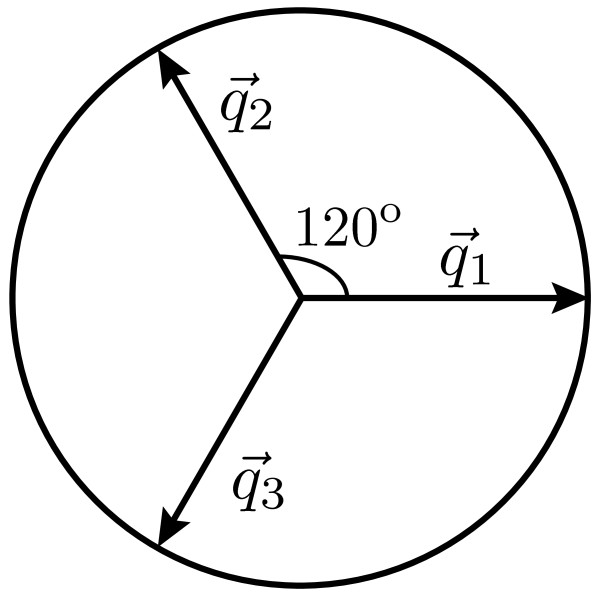
**Wave vectors of a hexagonal pattern.** Superposition of three wave vectors at an angle of 120 degree with each other to form a hexagonal pattern.

An application of the multiple-scale expansion [60] on the Brusselator model yields the following amplitude equation for the Turing mode [61]:

(26) $$\frac{\partial }{\partial t}Z_{1}=\mu Z_{1}+vZ_{2}^{*}Z_{3}^{*}-g{\left|Z_{1}\right|}^{2}Z_{1}-h\left({\left|Z_{2}\right|}^{2}+{\left|Z_{3}\right|}^{2}\right)Z_{1}$$

where

$$\begin{array}{ll} \mu =(B-B_{c})/B_{c}, & v=\frac{2}{A}\left(\frac{1-A\eta }{1+A\eta }\right)+\frac{2}{A}\mu ,\\ g=\frac{38A\eta +5{\left(A\eta \right)}^{2}-8-8{\left(A\eta \right)}^{3}}{9A^{3}\eta (1+A\eta )}, & h=\frac{5A\eta +7{\left(A\eta \right)}^{2}-3-3{\left(A\eta \right)}^{3}}{A^{3}\eta (1+A\eta )},\\ \eta =\sqrt{D_{X}/D_{Y}} & \end{array}$$

in which *B*_*c*_ is the critical value (see [54] for its setting) for Turing condition. Here, *Z*_(1,2,3)_ describes slow modulations of the Turing pattern, in short we call it Turing amplitude. The equations for *Z*_2_ and *Z*_3_ can be obtained from Eq. (26) by simple permutation of the indices. Indexed subscripts indicate that three amplitudes correspond to their respective wavevectors $\vec{q_{1}}$, $\vec{q_{2}}$ and $\vec{q_{3}}$ oriented at 120^o^ angular separations (see Figure 17). Ideally, the resonance of the three wavevectors result to a hexagonal or stripes modes. Practically, these modes may mix to form distorted patterns.

**Figure 18:**
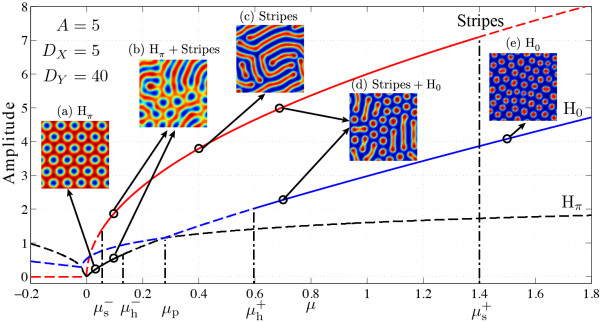
**Turing mode stability of the Brusselator model in 2-D space.** Each coloured stability curve represents specific mode: red = Stripes, blue = H_0_, black = H_*π*_. Solid and dashed curves correspond to stable and unstable modes respectively, according to mode stability analysis. Five representative *μ* values are selected for comparison of theoretical predictions for mode stability against practical simulations (shown as subplots). Colour of the pattern indicates the local concentration of the reactant: [red] high concentration, [blue] low concentration. Model parameters: *A* = 5,*D*_*X*_ = 5,*D*_*Y*_ = 40.

Following the work by Verdasca *et al.*[61], we applied linear stability analysis to the steady-state solutions of Eq. (26), leading to the pattern prediction diagram, shown in Figure 18. The diagram comprises of three stability curves revealing the stability of specific Turing modes: a honeycomb-like hexagonal structure, stripes, and reentrant honeycomb. Verdasca *et al.* denote the hexagonal mode as H_*π*_ and its reentrant form as H_0_ (see [61] for Verdasca *et al.*’s explanation on the subscripts *π* and 0). In this study, *μ* is a bifurcation control parameter, the value of which leads to a stable (solid curve) or unstable (dashed curve) mode.

For the 2-D spatiotemporal simulation of the Brusselator model, SIMULINK was set to 50-s simulation time in auto time-step mode, allowing the pattern-forming system to organise itself sufficiently. Figure 18 predicts the stabilities of stripes (red), H_0_ (blue) and H_*π*_ (black) modes for the Turing instability of the Brusselator model. From the range of solid curves, we have the summary of parametric space where a specific mode is stable: The stripes mode is stable when *μ*s- < *μ* < *μ*s+; the hexagonal mode is stable (H_*π*_ and H_0_) when *μ* < *μ*h- or *μ* > *μ*h+. H_*π*_ and H_0_ interact at *μ*_p_ where they exchange mode stability, that is, H_*π*_ will transit to H_0_ when *μ* crosses *μ*_p_ from its LHS to the RHS.

To verify the predictions from the bifurcation diagram, we select five different values of *μ* then examine the simulated patterns: 

(a) *μ* = 0.0495 falling into a range where only H_*π*_ is stable;

(b) *μ* = 0.1100 falling into a range where stripes and H_*π*_ modes coexist;

(c) *μ* = 0.3994 where only the stripes structure is stable;

(d) *μ* = 0.7000 again falling into a bistable range where stripes and H_0_ coexist;

(e) *μ* = 1.4802 where only H_0_ is stable.

In Figure 18, we see good agreement between SIMULINK simulated patterns and theoretical predictions. Clear H_*π*_, stripes and H_0_ structures are observed at (a), (c) and (e) cases respectively. (b) and (d) show mixed states between forward and backward stable structures. Consequently, we can conclude that by increasing *μ*, the distance to the critical point, positively, Brusselator forms sequentially from H_*π*_, to stripes, to H_0_.

In summary, to construct the Brusselator model in SIMULINK, we first place an integrator block to represent time derivative. The temporal integrator’s output will be fed back into the system to engage with the system’s evolution, then form the input of this integrator block, closing the loop. The model parameters can be adjusted by tuning the settings of the blocks *A* and *B* as well as two gains (labelled *D*_*X*_ and *D*_*Y*_ respectively). The real-time spatiotemporal evolution of *X* and *Y* are monitored via the Matrix viewer block. The simout block delivers the solution of Eq. (6) to the MATLAB workspace for future analysis. The solution is a three-dimensional matrix with the third dimension the same length as the time span.

In the next section, we will implement the SIMULINK-based cortical model and examine its clinically-relevant pattern dynamics.

### Cortical model stability and simulations

**Figure 19:**
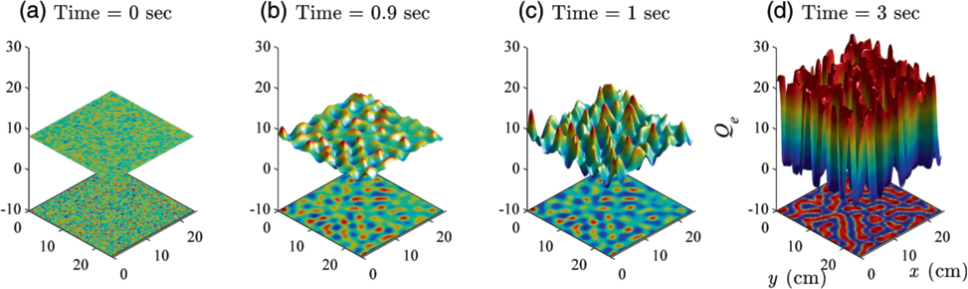
**Simulated cortical Turing patterns.** Four snapshots **(a–d)** taken from a 3-s simulation of the cortical Turing pattern of the excitatory firing-rate *Q*_*e*_ commencing from a homogeneous equilibrium. The top panel is the 3-D *Q*_*e*_ plot, and the bottom panel is the same information viewed on a bird’s-eye image. The gap-junction diffusity *D*_2_ = 1 cm^2^. The cortical model is initialised as a 100 × 100 grid with 20-cm side-length. SIMULINK is set to auto time-step mode. Simulation running time ∼30 s.

The work by Steyn-Ross *et al.*[44] suggests that the strong gap-junction diffusivity (large *D*_2_ value in the model equation (21)) provides a natural mechanism for Turing bifurcation that leads to the spontaneous formation of Turing labyrinth patterns of high and low neural activity that spread over the whole cortex, allowing multiple, spatially separated cortical regions to become activated simultaneously. Figure 19 illustrates the emergence of such cortical Turing patterns provoked by a strong inhibitory diffusion *D*_2_. It is possible that the Turing spatial synchrony explains the cognition “binding” phenomenon, which is, widely separated neural populations that are anatomically unconnected are in very similar states of activity, thereby becoming functionally connected and giving rise to coherent percepts and actions.

At the vicinity of a Turing instability, a weak Hopf instability can be induced in parallel by prolonging the timing of delivery of inhibition at chemical synapses. This permits slow Hopf oscillations with the spatial structure maintained. Specifically, a reduction of the inhibitory rate-constant *γ*_*i*_ in Eq. (20b) below a critical value ∼30.94 s^-1^ is sufficient to produce a complex dominant eigenvalue at zero wavenumber whose real part is positive; thus suggesting a global Hopf oscillation.

**Figure 20:**
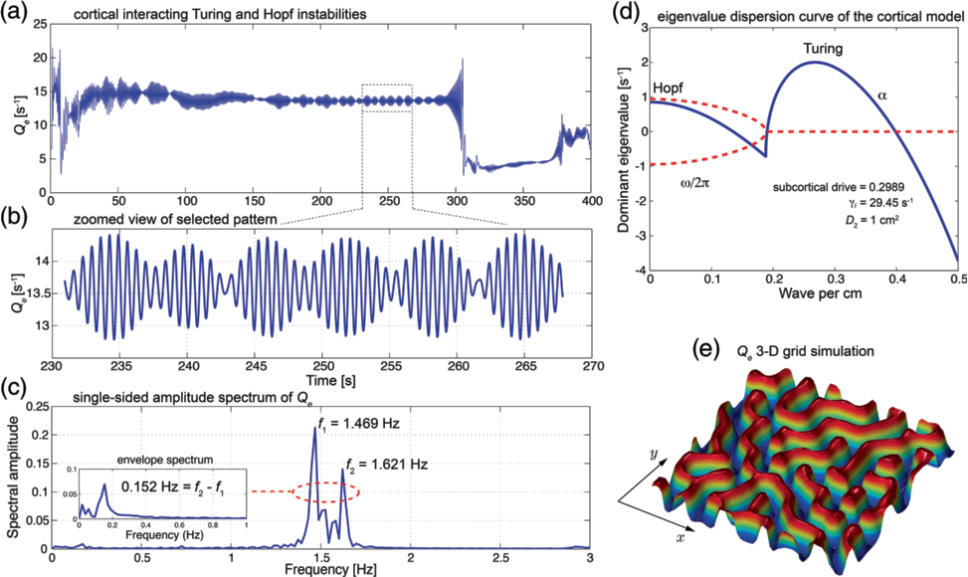
**Beating-wave patterns of the cortical model.** With strong gap-junction diffusion *D*_2_ and carefully chosen inhibitory rate-constant *γ*_*i*_, eigenvalue dispersion curve **(d)** predicts a mixed pattern of Turing and Hopf instabilities. *α* and *ω* are the real and imaginary part of the dominant eigenvalue respectively. Through a 400-s SIMULINK simulation, the Fourier spectrum **(c)** indicates a 0.15-Hz ultra-slow oscillation of the beating pattern **(b)** zoomed from **(a)** *Q*_*e*_ time evolution of the point at position (1, 30) shown in **(e)** 25- × 25-cm grid (100 × 100 grid-points) 3-D plot. Simulation running time ∼75 min. (Figure modified from [46]).

In Figure 20(d), setting *γ*_*i*_ = 29.45 s^-1^ predicts a ∼0.95 Hz Hopf oscillation. Independently, a Turing instability is boosted with moderately strong inhibitory diffusion *D*_2_ = 1 cm^2^ above its critical value 0.9066 cm^2^. Cortical Turing–Hopf interactions lead to beating patterns revealed in the time series recorded in Figure 20(b) for a single pixel on the cortical grid. The Fourier spectrum shows two frequency components whose difference matches with the ultra-slow envelope frequency, which is likely to be the weakly-damped resonance at ∼0.152 Hz.

Steyn-Ross *et al.*[46] posit that the interacting low-frequency Hopf and Turing instabilities may form the substrate for the cognitive state, namely, the “default” background state for the non-cognitive brain. These slow patterned oscillations may relate to very slow (≤0.1 Hz) fluctuations in BOLD (blood-oxygen-level dependent) signals detected using fMRI (functional magnetic resonance imaging) of relaxed, non-tasked human brains [62,63]. Steyn-Ross *et al.*[47] also predict that this default state will be suppressed with elevated levels of subcortical drive during goal-directed tasks [64-68].

### The effect of embedding MATLAB functions in SIMULINK running efficiency

Although SIMULINK has an intuitive programming logic and comparable accuracy to MATLAB, it sometimes runs much slower than MATLAB, e.g., in the demonstrated Brusselator and cortical model simulations. The reason is that we embed MATLAB functions wraparound in the model to expand the SIMULINK capability. Once a MATLAB function block is present, the MATLAB interpreter is called at each time-step. This drastically reduces the simulation speed. So, one should use the built-in blocks whenever possible. Without using MATLAB function blocks, SIMULINK shows a higher performance than MATLAB, e.g., see the description of Figure 3. MathWorks Support Team also presented comprehensive guidance to speed up the SIMULINK simulation, which are available at http://www.mathworks.com/matlabcentral/answers/94052. In the further optimisation of our SIMULINK model, we will consider replacing MATLAB function with the MEX S-function, which may help to accelerate the simulation in the merit of its direct communication with the SIMULINK engine (avoid the time consuming compile-link-execute cycle). The build of MEX S-function requires higher level programming skills, so we only address a more accessible method (embedding MATLAB function) for non-experts in this paper.

## Conclusions

The strategy for modelling differential equation models in SIMULINK is addressed in this paper. To construct a system of differential equations in SIMULINK, we can directly convert the mathematical terms and operators to the SIMULINK graphical block diagrams. The key idea for programming differential systems in SIMULINK is to form a closed loop, such that the solution of the system can evolve in this loop. The accuracy and reliability of the SIMULINK modelling method has been examined via comparing a van der Pol oscillator represented in MATLAB code-script and SIMULINK block-diagram, showing the SIMULINK model has comparable performance with the code-script version.

Using a well-known Brusselator model, we demonstrated two main SIMULINK modelling strategies for a reaction-diffusion system: interpretation of 2-D convolution with the periodic boundary condition; hybrid programming in SIMULINK with MATLAB functions. The pattern simulations of the Brusselator in SIMULINK are in good agreement with the predictions via bifurcation theories.

Following the pattern-forming theories, we introduced a cortical model with competitive neuronal interactions and diffusions. Unlike the simple Brusselator, the cortical model is a complicated system comprised of distinct cortical connections. Here, we showed how to build these subsystems in SIMULINK. Finally, we connected all subsystems to form the completed Waikato cortical model. The simulations of the cortical model exhibit Turing and mixed Turing–Hopf patterns, the clinical relevance of which is briefly discussed. As the main aim of this paper is to introduce SIMULINK modelling, readers are referred to Steyn-Ross *et al.*’s recent publications [6,44-47] for comprehensive investigations of the cortical model.

## Endnotes

^a^vanderpoldemo is a MATLAB pre-coded function

^b^fixed time-step ODE solvers are not built into MATLAB, but they can be acquired from a release by the MathWorks Support Team: http://www.mathworks.com/matlabcentral/answers/uploaded_files/5693/ODE_Solvers.zip

^c^The two-dimensional circular convolution algorithm was written by David Young, Department of Informatics, University of Sussex, UK. His convolve2() code can be downloaded from MathWorks File Exchange: http://www.mathworks.com/matlabcentral/fileexchange/22619-fast-2-d-convolution

^d^MATLAB simulation codes were written by Alistair Steyn-Ross. The complete codes, plus README files and movies of cortical dynamics, are available from the web site: http://www2.phys.waikato.ac.nz/asr/

## Competing interests

The authors declare that they have no competing interests.

## Authors’ contributions

All authors contributed extensively to the work presented in this paper: MSR, ASR, MTW and JWS developed the cortical model; MSR proposed the stability analysis; KW conceived of Simulink modelling for reaction-diffusion systems and conducted the conversion for the Brusselator and cortical models; ASR provided guidance for the dimensional scaling of the Simulink spatial grids; YS participated in the Simulink modelling; all authors contributed to the writing of the paper; all authors read and approved the final manuscript.
